# Recent Advances in Immunotherapy in Metastatic NSCLC

**DOI:** 10.3389/fonc.2016.00239

**Published:** 2016-11-14

**Authors:** Pranshu Bansal, Diaa Osman, Gregory N. Gan, George R. Simon, Yanis Boumber

**Affiliations:** ^1^Department of Internal Medicine, Division of Hematology/Oncology, University of New Mexico Comprehensive Cancer Center, University of New Mexico School of Medicine, Albuquerque, NM, USA; ^2^Hematology/Oncology Fellowship Program, University of New Mexico Comprehensive Cancer Center, University of New Mexico School of Medicine, Albuquerque, NM, USA; ^3^Section of Radiation Oncology, University of New Mexico Comprehensive Cancer Center, Albuquerque, NM, USA; ^4^Department of Thoracic and Head/Neck Medical Oncology, Division of Cancer Medicine, University of Texas MD Anderson Cancer Center, Houston, TX, USA; ^5^Molecular Therapeutics Program, Department of Hematology/Oncology, Fox Chase Cancer Center, Philadelphia, PA, USA

**Keywords:** PDL1, PD1, CTLA4, NSCLC, cancer immunotherapy

## Abstract

Non-small cell lung cancer (NSCLC) is one of most common malignancies and the leading cause of cancer deaths worldwide. Despite advances in targeted therapies, majority of NSCLC patients do not have targetable genomic alterations. Nevertheless, recent discovery that NSCLC is an immunogenic tumor type, and several breakthroughs in immunotherapies have led to rapid expansion of this new treatment modality in NSCLC with recent FDA approvals of programed death receptor-1 inhibitors, such as nivolumab and pembrolizumab. Here, we review promising immunotherapeutic approaches in metastatic NSCLC, including checkpoint inhibitors, agents with other mechanisms of action, and immunotherapy combinations with other drugs. With advent of immunotherapy, therapeutic options in metastatic NSCLC are rapidly expanding with the hope to further expand life expectancy in metastatic lung cancer.

## Immunotherapy in Cancer: Introduction

In 1957, Sir Macfarlane Burnet and Lewis Thomas first recognized the antigenicity of tumors and discovered immune surveillance in tumorigenesis (1). However, translation of the use of immune system for clinically meaningful benefits has started to realize only recently.

Compared to the circulating tumor antigen-specific T-cells, T-cells in the tumor microenvironment have impaired effector functions (2–4). Mice infected with chronic lymphocytic choriomeningitis virus (LCMV) show upregulation of negative costimulatory receptors on T-cells, including cytotoxic T-lymphocyte antigen 4 (CTLA-4), programed death receptor-1 (PD-1), T-cell immunoglobulin domain and mucin domain-3 (TIM-3), lymphocyte activation gene-3 protein (LAG-3), and others (5). The loss of T-cells effector function during chronic infections is similar in cancer where T-cells chronically exposed to tumor antigens enter a state of exhaustion (6). Major efforts concentrating on reversing T-cell exhaustion by inhibiting negative checkpoints with antibodies against PD-1 and its ligand, programed death receptor-1 ligand (PD-L1) are at the forefront of immunotherapy research.

Clinical success with immunotherapy was first seen in highly immunogenic malignant melanoma. High-dose interleukin-2 (IL-2) showed an objective response rate (ORR) of 16% (7). Interestingly, many complete responders experience long-term responses, and IL-2 was used extensively in melanoma patients despite high toxicities (8). This changed with approvals of CTLA-4 inhibitor ipilimumab in 2011, and PD-1 inhibitors, such as pembrolizumab and nivolumab, in 2014, which have better side effect profiles. The response rates with ipilimumab are ~10%, while PD-1 inhibitors have ~40% ORR (8, 9). Combination studies of ipilimumab and nivolumab have shown ORR of 42–60% (9, 10), unprecedented in melanoma, have led to survival benefits and safer treatment options.

Lung cancer has been viewed as non-immunogenic tumor where immunotherapies, such as BCG and IL-2, showed no efficacy (11, 12). However, with recent success of immune checkpoint inhibitors, this has changed, and here we review the recent data in non-small cell lung cancer (NSCLC) and emerging immunotherapies.

## Established Single-Agent Checkpoint Inhibitors

### Nivolumab

Nivolumab is a fully human IgG4 antibody against PD-1, which can activate host immune system. In phase I studies in pretreated NSCLC patients, nivolumab showed ORR of 17–18% (13, 14). A phase III trial in patients with advanced squamous NSCLC compared nivolumab with docetaxel, demonstrating median OS 9.2 vs. 6.2 months (15). In another phase III trial, patients with advanced non-squamous NSCLC progressing on platinum-doublet chemotherapy were randomized to receive nivolumab or docetaxel, showing median OS 12.2 months with nivolumab and 9.4 months with docetaxel (16). The subgroup of patients without numerical progression-free survival (PFS) advantage with nivolumab included never smokers, patients with KRAS wild type, EGFR-mutant tumors, although this study was not powered for subgroup analysis. PD-L1 expression was detected by IHC using a human PDL-1 antibody (Epitomics). Cutoffs were divided into three groups: 1, 5, and 10%. Quantifiable PDL-1 expression was seen in 78% patients. OS difference favoring nivolumab was found to be significant across all subgroups. No meaningful advantage in survival was seen in patients with PDL-1-negative tumors (16). FDA approved nivolumab for NSCLC patients progressing on platinum-doublet chemotherapy in 2015.

The role of nivolumab in patients with Eastern Cooperative Oncology Group performance status 2, as maintenance therapy, and in combination with other therapies remains to be established. A five-arm, phase II basket study has been launched with ~2000 NSCLC patients including PS2 patients, maintenance cohorts, and epidermal growth factor receptor positive (EGFR+)/anaplasplastic lymphoma kinase positive (ALK+) patients treated with erlotinib or crizotinib with and without nivolumab (CHECKMATE 370; NCT02574078).

### Pembrolizumab

Pembrolizumab is another monoclonal antibody targeting PD-1. A randomized phase II/III study compared pembrolizumab with docetaxel in advanced NSCLC patients (70% non-squamous, 22% squamous histology), excluding those with PD-L1-negative tumors and stratifying by PDL-1 expression 1–49% (~57% patients) and >50% (~43%). The ORR with pembrolizumab was ~18 vs. 9% with docetaxel, similar to nivolumab studies. The ORR in patients with >50% PD-L1 expression was higher: ~30 vs. 8% with docetaxel. Median OS was 14.9 months for the pembrolizumab 2 mg/kg, 17.3 months for the pembrolizumab 10 mg/kg, and 8.2 months for the docetaxel group (17). FDA approved pembrolizumab in 2015 for patients with PDL1 + NSCLC patients with mandatory immunohistochemistry PD-L1 diagnostic test 22C3 (Dako).

### Atezolizumab

Programed death receptor-1 has two ligands: PD-L1 (B7-H1) and PD-L2 (B7-DC) (18, 19). PD-L1 is expressed on hematopoietic, endothelial, and epithelial cells (20). Atezolizumab is an IgG1 antibody to PD-L1. In a phase II study (POPLAR) of NSCLC patients progressing on platinum-based chemotherapy, atezolizumab was compared with docetaxel. The median OS for atezolizumab was significantly better than with docetaxel (12.6 vs. 9.7 months, *p* = 0.04). The improvement correlated with PD-L1 expression on both tumor and tumor-infiltrating immune cells. Although the ORR was 15% for both arms, subgroup of patients with high PD-L1 expressing tumors had ORR of 38% with atezolizumab and 13% with docetaxel (21). In another large single-arm phase II study (BIRCH), PD-L1+ patients included treatment-naïve NSCLC patients in cohort 1; cohorts 2/3 included patients after 1–2 lines of chemotherapy. The ORR was 19% in cohort 1, 17% in cohorts 2/3. PD-L1 positivity was defined as >5% tumor cells positive or >5% tumor area with immune cells staining positive (22). FDA has granted priority review for atezolizumab, and final decision approval is expected on October 2016.

### Durvalumab

Similar to atezolizumab, durvalumab is an IgG1 antibody to PD-L1. In a phase I study, durvalumab was administered to patients with NSCLC progressing after chemotherapy. Among 149 patients, ORR was 14% favoring PD-L1+ patients (ORR 23%) and squamous (ORR 21%) compared to non-squamous histology (ORR 10%) (23). In treatment-naive patients with advanced NSCLC, ORR was 25% in a recent phase I/II study (24). ATLANTIC is an ongoing phase II study with durvalumab in NSCLC patients progressing after at least two prior chemotherapies (NCT02087423).

### Avelumab

Similar to durvalumab, avelumab is a fully human IgG1 antibody against PDL1. In a phase Ib study, 184 NSCLC patients progressing on platinum-based chemotherapy, avelumab showed an ORR of 12% (14% in PD-L1 + population) (25). JAVELIN lung 100 is an ongoing phase III trial evaluating avelumab vs. platinum-based doublet chemotherapy in treatment-naïve NSCLC patients (NCT02576574). JAVELIN lung 200 is a phase III trial in patients progressing after platinum-based chemotherapy, comparing avelumab to docetaxel (NCT02395172).

### PD-1, PD-L1 Targeting Agents: Selecting the Right Patient Population

Scagliotti et al. first demonstrated difference in survival in NSCLC patients treated with platinum-doublet chemotherapy based on histology, a biomarker of chemotherapy response. Squamous histology patients did better with cisplatin/gemcitabine, while adenocarcinoma patients had improved OS on cisplatin/pemetrexed combination (26).

Predicting responses to immunotherapy is more complicated; PD-L1 expression is one potential marker. PD-L1 expression can change over time. PD-L1 expression at initial tumor biopsy does not always correlate with antitumor activity (27). Thommen et al. evaluated expression of checkpoints on CD8+ T-cells from patients with advanced NSCLC and developed a cumulative inhibitory score based on sum expression of several receptors. Significant increase in the score was seen in patients with node positive, advanced stage, while tumor size did not affect it. An inverse correlation between PDL-1 expression and effector function of CD8+ T-cells treated with PD-1 antibody was observed (28). While PD-L1 expression is one of the markers for response in NSCLC, prior smoking and high mutational load correlate with responsiveness, suggesting that better predictive biomarkers or scores are needed (29). Schumacher et al. developed cancer immunogram with seven different immune parameters including mutational load to help predict responsiveness of tumors to checkpoint inhibitors (30). Efforts should be focused in this direction to help identifying the target population and to drive down treatment costs.

### Ipilimumab and Tremelimumab

Ipilimumab and tremelimumab are recombinant monoclonal antibodies against cytotoxic CTLA-4 that block CTLA-4 interaction with its ligands, such as CD80 and CD86. This blockade augments T-cell activation and proliferation, leading to tumor infiltration by T-cells and tumor regression (31). Although response rates with single-agent CTLA-4 inhibitor in NSCLC have been disappointing, there is considerable interest in exploiting this pathway in combination with other checkpoint inhibitors (32). In a phase II study, ipilimumab in combination with chemotherapy showed modest improvement in PFS in patients with advanced NSCLC (33). The benefit was limited to patients who received phased ipilimumab, allowing exposure to chemotherapy before ipilimumab (33). Encouraging results seen in early stage combination trials of CTLA-4 with PD-1 and PD-L1 inhibitors are discussed below.

### Epigenetic Approaches to Improve Immunotherapy Responses

Epigenetic silencing and loss of gene expression is a hallmark of cancer (34). DNA-demethylating and histone-deacetylating agents show modest benefit in solid tumors (35, 36). Some patients may have durable response to subsequent chemotherapy or immunotherapy after receiving epigenetic modulators (37). Epigenetic therapy could sensitize tumors to subsequent therapy by altering tumor genomics, increasing tumor antigenicity (35, 38, 39). In a phase I/II trial, heavily pretreated NSCLC patients received AZA and etinostat. Among 65 patients treated in phase II portion, 2 responded (37). Six patients from this trial subsequently received PD-1 inhibitors and five of the six patients showed PR, including three patients with durable responses for 14–26 months (40).

Strong preclinical rationales for the combining epigenetic drugs with immunotherapy remains, including induction of IRF-7 gene, secretion of INF-alpha, synergy between AZA and CTLA4, and others (38, 40). Ongoing studies are evaluating potential combination of demethylating agents and HDAC inhibitors with nivolumab, pembrolizumab, and durvalumab, including patients failing prior PD-1/PDL-1 therapy (NCT01928576, NCT02638090, NCT02437176, NCT02437136, NCT02805660).

## Emerging Single-Agent Checkpoint Inhibitors

### T-Cell Immunoglobulin and Mucin Domain-3-Containing Molecule 3

With the success of PD-1/PDL-1 and CTLA-4 inhibitors in NSCLC, there is an increase interest in exploring other immune checkpoint regulators and understanding mechanisms of resistance to checkpoint inhibitors. Ectopic expression of TIM-3 was previously reported as an independent negative prognostic factor in NSCLC (2). Upregulation of alternative immune checkpoints, notably TIM-3, is implicated in PD-1 inhibitors resistance (39). Hammerman et al. showed upregulation of TIM-3 in PD-1 antibody-bound T-cells and a survival advantage in mice lung adenocarcinoma xenografts with addition of TIM-3 antibody to PD-1 inhibitor. They found similar upregulation of TIM-3 in biopsies from patients progressing on PD-1 therapy (41). Currently, the first in humans phase I-Ib/II trial is ongoing with MBG453 antibody against TIM-3 in combination with PDR001 PD-1 inhibitor (NCT02608268) in patients with advanced solid malignancies, including NSCLC.

### Lymphocyte Activation Gene-3

Lymphocyte activation gene-3 is a transmembrane protein, closely related to CD4 that binds major histocompatibility complex II (42). LAG-3 is co-negative regulator of effector T cells (43). Regulatory T-cells (Tregs) express LAG-3 that is upregulated in the presence of effector T-cells, leading to decreased antitumor immunity (44). LAG-3 antibodies inhibit Tregs (44). Preclinical data in multiple tumors demonstrated prolonged survival with dual PD-1/LAG-3 inhibition (45). Tissue samples from patients with ovarian epithelial cancers tumor-derived T-lymphocytes showed enrichment for coexpression of LAG-3 and PD-1 (46). Most of LAG-3 data at this time remain preclinical, and efficacy in solid malignancies remains to be seen. Urelumab (BMS-986016) and LAG525 are anti-LAG-3 antibodies investigated in early phase clinical trials in solid tumors including NSCLC alone and in combination with nivolumab or PDR-001 (NCT02658981, NCT01968109, NCT02460224).

## Emerging Single-Agent Immunomodulating Drugs

### Bavituximab

Bavituximab is a chimeric immunoglobulin antibody targeting a common phospholipid, phosphatidyl serine (PS), on tumor vascular endothelium (47). In normal cells, PS is expressed on internal membrane surface; however, in tumors, it becomes exposed on endothelial cells. Bavituximab binds to PS in a β2-glycoprotein I-dependent manner leading to antitumor effects such as antibody-dependent cell-mediated toxicity. Bavituximab prevents interaction between PS on apoptotic tumor cells and macrophages and decreases secretion of anti-inflammatory immunosuppressive molecules, such as TGF-β and IL-10 (48, 49).

In a phase II trial, docetaxel + bavituximab (D + B) was compared to docetaxel + placebo (D + P) in patients with locally advanced lung adenocarcinoma. The D + B combination was well tolerated, and results were encouraging with an improvement of ORR from 11.3% (D + P arm) to 17.1% (D + B) (50). The confirmatory phase III SUNRISE trial was halted when at 33% events it failed to show OS advantage of docetaxel + bavituximab combination (NCT01999673). A combination immunotherapy trial with durvalumab (PD-L1 inhibitor) and bavituximab in recurrent NSCLC is ongoing (NCT02673814).

### Indoleamine 2,3-Deoxygenase

Indoleamine 2,3-deoxygenase (IDO) is a cytoplasmic tryptophan catabolic enzyme that plays a significant role in T-cell suppression (51). High levels of IDO were first reported in human placenta (52). Inhibition of IDO in mice placenta leads to T-cell-mediated rejection of allogeneic conceptii (53). In addition to promoting immune tolerance to fetus in placenta, IDO-mediated depletion of tryptophan prevents excessive T-cell activation and lung inflammation (54). IDO supports development of immune tolerance to tumor antigens, activation of regulatory T-cells, and inhibition of cytotoxic T-cells and NK cells and is a relevant target in tumor immunology (55).

An ongoing trial is evaluating a combination of IDO inhibitor indoximod with docetaxel and Tergenpumatucel-L in NSCLC (NCT02460367). Ongoing basket trials in solid malignancies including NSCLC are testing other IDO inhibitors alone or in combination with checkpoint inhibitors (NCT02048709, NCT02073123).

## Emerging PD1/PDL1 Immunotherapy Combinations with CTLA4 Inhibitors, Chemotherapy, and Targeted Therapy

Immunotherapy combinations hold promise to further increase ORR and OS and outperform chemotherapy in NSCLC in the first line settings, based on success of nivolumab/ipilimumab combination in metastatic melanoma, now FDA approved based on CheckMate067 trial results (56).

Multiple studies are evaluating the role of frontline immunotherapy in treatment-naïve NSCLC patients. CheckMate012 trial included 148 treatment-naïve NSCLC patients with PDL-1 expression >1%; 80% patients had non-squamous histology. Arm A patients received nivolumab and ipilimumab Q3 weeks with ORR 13%, arm B – nivolumab 1 mg/kg Q2 weeks, ipilimumab 1 mg/kg Q6 weeks, ORR was 25%. Arm C had the best ORR of 39% when nivolumab was given 3 mg/kg Q2 weeks and ipilimumab 1 mg/kg Q12 weeks. Arm D had an ORR 31% – nivolumab Q2 weeks, ipilimumab Q6 weeks. Median duration of response was not reached (57). A phase II single-arm ipilimumab–nivolumab trial in metastatic NSCLC is underway (CheckMate 568, NCT02659059). Important ongoing phase III studies that might change the treatment landscape of NSCLC are KEYNOTE-024 comparing pembrolizumab to chemotherapy in PD-L1 + NSCLC, and CheckMate 026 with nivolumab, both in treatment-naïve advanced NSCLC patients.

CheckMate 227, a phase III ipilimumab–nivolumab study in treatment-naïve advanced NSCLC in comparison to chemotherapy, is ongoing (NCT02477826). Combination of PD-L1 inhibitor durvalumab and CTLA4 inhibitor tremelimumab was recently studied in a phase Ib study of 102 pretreated NSCLC patients. Significant dose-limiting toxicities were seen in durvalumab 20 mg/kg, tremelimumab 3 mg/kg cohort. Encouraging ORR of 23% irrespective of PD-L1 status was observed in patients treated with durvalumab 20 mg/kg and tremelimumab 1 mg/kg (24). MYSTIC and NEPTUNE are ongoing phase III studies comparing this combination to platinum-based chemotherapy in treatment-naïve NSCLC patients (NCT02453282, NCT02542293).

Immunotherapy was pioneered in relatively chemoresistant tumors, melanoma, and renal cancer. Historically, platinum-doublet chemotherapy in NSCLC has ORR ~30% with median OS ~11–13 months (26, 58, 59). Addition of bevacizumab had modest effect on OS (60). Immunotherapy success in NSCLC opened possibilities of combining PD1/PDL1 inhibitors with platinum-based doublet chemotherapies in a relatively chemosensitive tumor. KEYNOTE-021 is one such trial (61). Treatment-naïve stage IIIB/IV NSCLC patients were randomized to pembrolizumab in combination with carboplatin/paclitaxel (cohort A), carboplatin/paclitaxel/bevacizumab (cohort B), and carboplatin/pemetrexed (cohort C). ORR in cohort C was 71% compared with cohort A (52%). Similar ORR was seen in patients expressing <1% PD-L1. The carboplatin/pemetrexed/pembrolizumab combination is now tested in a larger study (NCT02039674). Another phase III trial KENOTE-189 is comparing platinum-doublet ± pembrolizumab in advanced NSCLC patients (NCT02578680).

In EGFR or ALK-positive NSCLC, several early phase trials are ongoing, combining PD-1 and CTLA-4 checkpoint inhibitors with EGFR (erlotinib, afatinib, osimertinib) or ALK (crizotinib) tyrosine kinase inhibitors (TKIs) in treatment-naive and previously treated patients (NCT01998126, NCT02511184). TATTON is a multi-arm phase Ib trial of durvalumab with osimertinib. Dose escalation arm included EGFR TKI-naïve patients, while the expansion cohort included patients progressing on prior TKIs. Although the combination showed clinical activity, 38% rate of interstitial lung disease was reported with the combination, disproportionate to what is expected with each agent alone. The combination arm has been put on hold (62). A phase I feasibility trial of afatinib and pembrolizumb for patients with EGFR mutation-positive NSCLC progressing on erlotinib is recruiting (NCT02364609).

### Immune-Mediated Toxicity

Immunotherapy is associated with increased frequency of immune-related side effects, a spectrum of side effects that are broadly termed immune-related adverse events (irAEs) (63). irAEs with PD-1/PD-L1 inhibitors are less common (7–13%) compared to toxicities with CTLA4 inhibitors (10–18%) (15–17, 63, 64).

The most frequent side effect with PD-1/PD-L1 inhibitors is fatigue, reported among 14–32% patients in phase III studies (17–19, 23). Organ-specific common side effects include skin toxicities such as rash (9%), diarrhea/colitis (7–8%), hypothyroidism (7–8%), hepatitis (1–3%), adrenal insufficiency (1%), and thyroiditis (1%) (17–19). Grade 3/4 pneumonitis has been reported in NSCLC (3%) with rare treatment-related deaths from pneumonitis (64, 65).

Frequency of irAE from combination immunotherapies is higher. In phase Ib study of treatment-naïve NSCLC patients receiving durvalumab and tremelimumab, serious adverse events were reported in 36% patients. The most frequent grade 3/4 toxicities were diarrhea (11%), colitis (9%), and increased lipase (8%) with three treatment-related deaths (24). Toxicity analysis from multi-cohort CheckMate 012 showed 29–35% grade 3/4 toxicity in nivoluamb plus ipilimumab combination arms compared to 19% grade 3/4 toxicity in the nivolumab-only arm (57, 66).

## Conclusion

**Figure 1:**
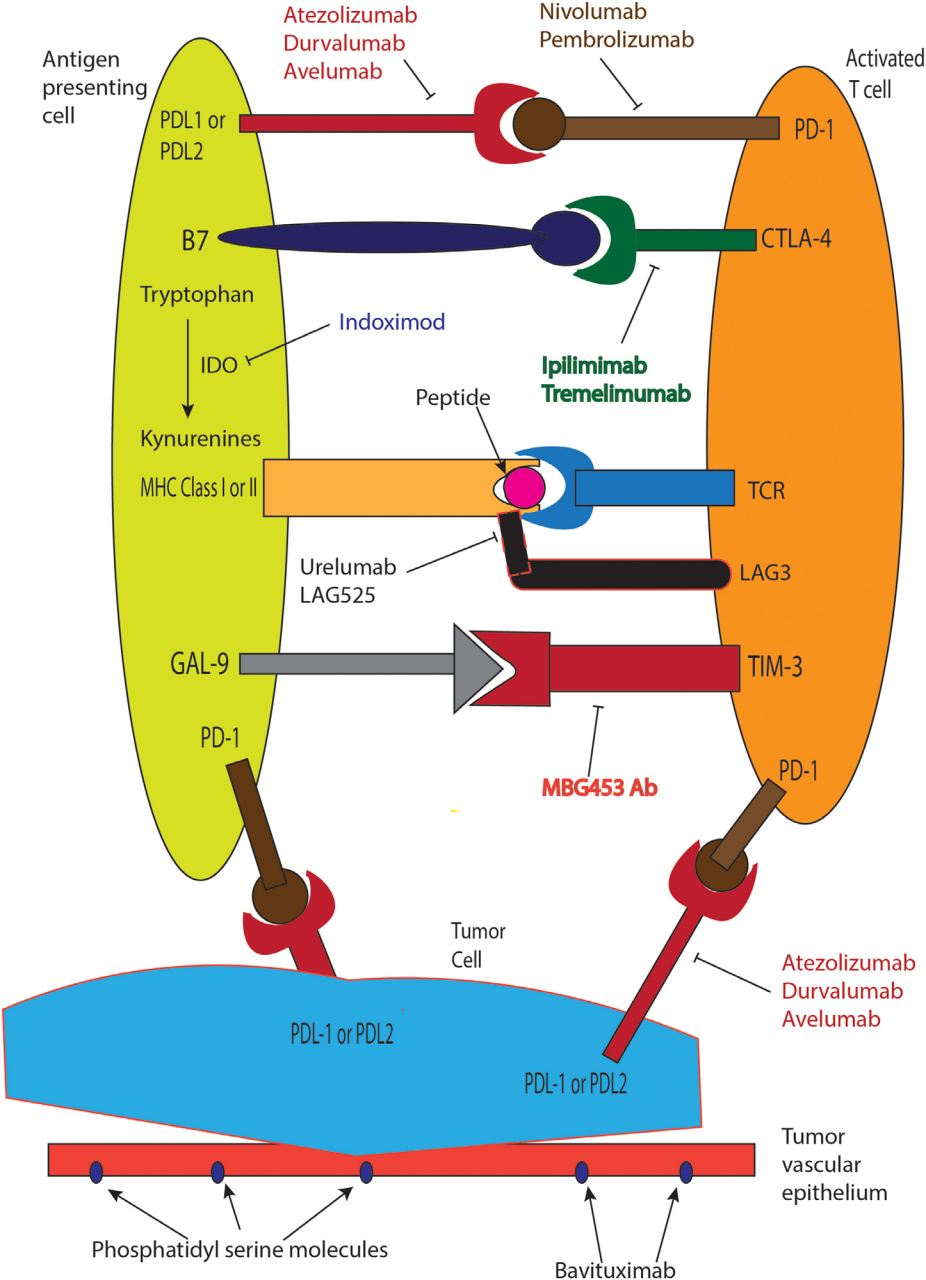
**Mechanism of action of clinically used immunotherapeutic agents**.

Advances in immunotherapy in metastatic NSCLC now include nivolumab and pembrolizumab that are standard in second line settings, where both agents significantly improve survival. The treatment paradigm changed with introduction of immunotherapy and is expected to start impacting first-line treatment approaches soon. Ongoing challenges include understanding mechanisms of resistance to immunotherapy drugs, development of biomarkers, and predictive scores for better patient selection. Table 1 summarizes ongoing immunotherapy trials in advanced NSCLC, and Figure 1 describes mechanisms of action of select agents. New immunotherapy drugs and novel immunotherapy combinations in frontline and recurrent settings in NSCLC remains one of the most exciting and rapidly evolving areas in oncology, with hope to increase the numbers of long-term survivors with stage IV lung cancer in the near future.

**Table 1:** **Selected immunotherapy studies in metastatic NSCLC**.

Experimental arm	Comparator arm	Patient population	Trial stage	Primary end point (trial identification number)
Nivolumab vs. nivolumab + ipilimumab vs. nivolumab + platinum-doublet chemotherapy (CHECKMATE 227)	Platinum-doublet chemotherapy	Treatment naïve stage IV NSCLC	III	Overall survival progression-free survival (NCT02477826)
Nivolumab + ipilimumab (CHECKMATE 568)	None-single arm	Treatment naïve stage IV NSCLC	II	Overall response rate (ORR) (NCT02659059)
Nivolumab or ipilimumab + erlotinib (EGFR mutant) or crizotinib (ALK mutant)	None-single arm	Stage IV NSCLC failed prior platinum-based therapy	I	Toxicity of nivolumab or ipilimumab in combination with erlotinib (EGFR-mutant NSCLC) or crizotinib (ALK mutant NSCLC)
Nivolumab + ALT 803 (novel recombinant IL-15 complex)	None	Stage IIIb/IV NSCLC failed prior platinum-based therapy	Ib/II	Phase Ib – dose-limiting toxicity of ALT 803 nivolumab combination
				Phase II – ORR (NCT02523469)
Pembrolizumab + vorinostat	None	Stage IV NSCLC progressed on at least one prior therapy	Ib/II	Phase Ib – maximum tolerated dose
				Phase II – ORR (NCT02638090)
Pembrolizumab (KEYNOTE-024)	Doublet chemotherapy	Treatment naïve stage IV NSCLC with strongly expressing PD-L1	III	PFS (NCT024142738)
Multi-arm pembrolizumab plus chemotherapy or immunotherapy (KEYNOTE-021)	None	Treatment naïve stage IV NSCLC	I/II	Part I – recommended phase II dose of pembrolizumab
				Part II – ORR (NCT02039674)
Durvalumab vs. durvalumab plus tremelimumab (MYSTIC)	Standard of care platinum-doublet chemotherapy	Treatment naïve stage IV NSCLC	III	OS and PFS (NCT02453282)
Durvalumab plus tremelimumab (NEPTUNE)	Standard of care platinum-doublet chemotherapy	Treatment naïve stage IV NSCLC	III	OS (NCT02542293)
Atezolizumab + carboplatin/paclitaxel ± bevacizumab	Caboplatin + paclitaxel + bevacizumab	Treatment naïve NSCLC – non-squamous histology	III	PFS (NCT02366143)
Atezolizumab	Gemcitabine + ciplatin or carboplatin	Treatment naïve NSCLC – squamous histology	III	PFS (NCT02367794)
MGB453 (anti TIM-3) plus PDR001 (anti PD-1)	None	Multiple advanced solid malignancies including NSCLC progressed on standard therapy	I–Ib/II	Safety and tolerability of MGB453 in combination with PDR001
				ORR (NCT02608268)
LAG525 (anti LAG-3) single agent and in combination with PDR001 (anti PD-1)	None	Multiple advanced solid malignancies including NSCLC progressed on standard therapy	I/II	Phase I-dose-limiting toxicity
				Phase II-ORR

## Author Contributions

First author – PB – prepared manuscript and table. Corresponding author – YB – prepared part of the manuscript and table and provided guidance to the first author in preparing the manuscript; also helped in editing the article and the figure. Co-author – DO – prepared the figure. Co-author – GS – helped with review and vital modifications along with suggestions to improve the content. Co-author – GG – helped with review and edition along with suggestions to improve the content.

## Conflict of Interest Statement

The authors declare that the research was conducted in the absence of any commercial or financial relationships that could be construed as a potential conflict of interest.
